# Supplementary material for: Long-read sequencing of families reveals increased germline and postzygotic mutation rates in repetitive DNA
Source: Nat Commun. 2026 Mar 9;17:3717. doi: 10.1038/s41467-026-70342-1 (PMC13102968; doi:10.1038/s41467-026-70342-1)
Supplement: Supplementary file 1 — Supplementary Information [file 41467_2026_70342_MOESM1_ESM.pdf]

Long-read sequencing of families reveals increased germline and postzygotic mutation rates in repetitive DNA

Supplementary Figures

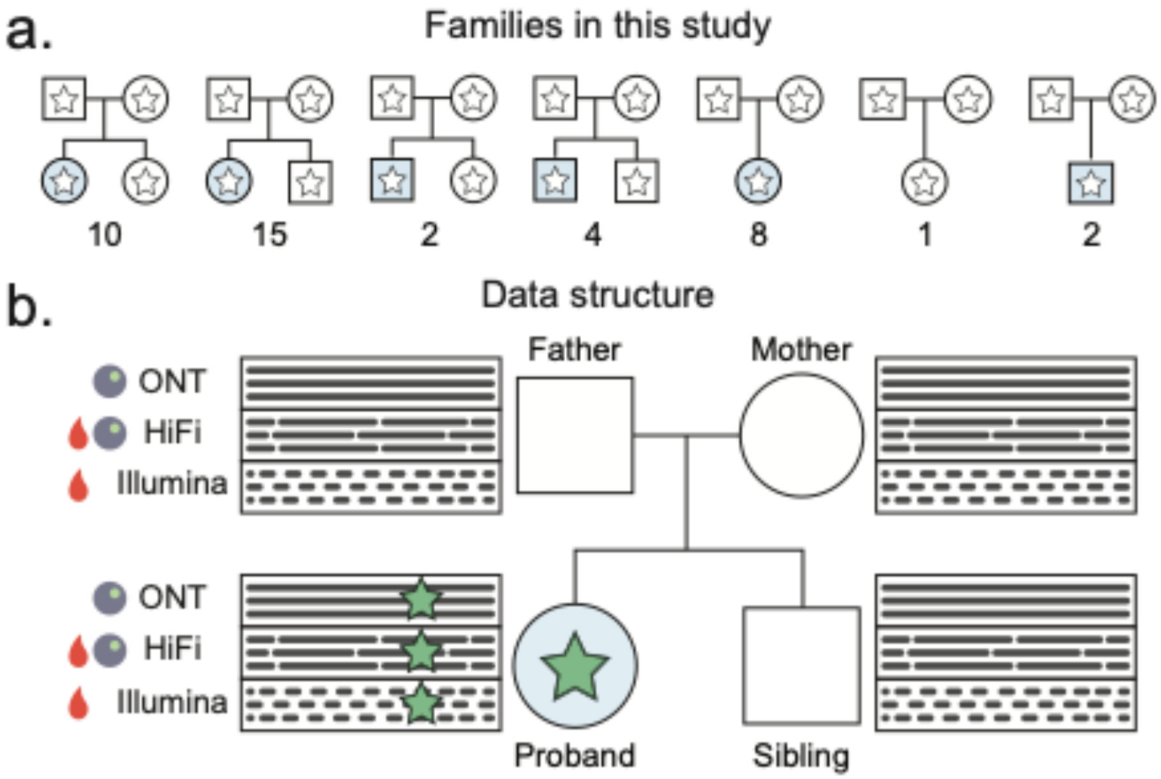

Supplementary Figure 1: Overview of study design

a. Family pedigrees, including 31 quads and 11 trios. Note that one family has a sibling but not an ASD-affected proband; this was due to lack of proband data availability at time of study.

b. We sequenced all three (trio) or four (quad) members with three sequencing platforms: Oxford Nanopore Technologies (ONT) on cell-line-derived DNA (n=73/74 children), PacBio HiFi on blood-derived DNA (n=64/73 children) and cell-line-derived DNA (n=25/73 children), and Illumina on blood-derived DNA (n=73/73 children).

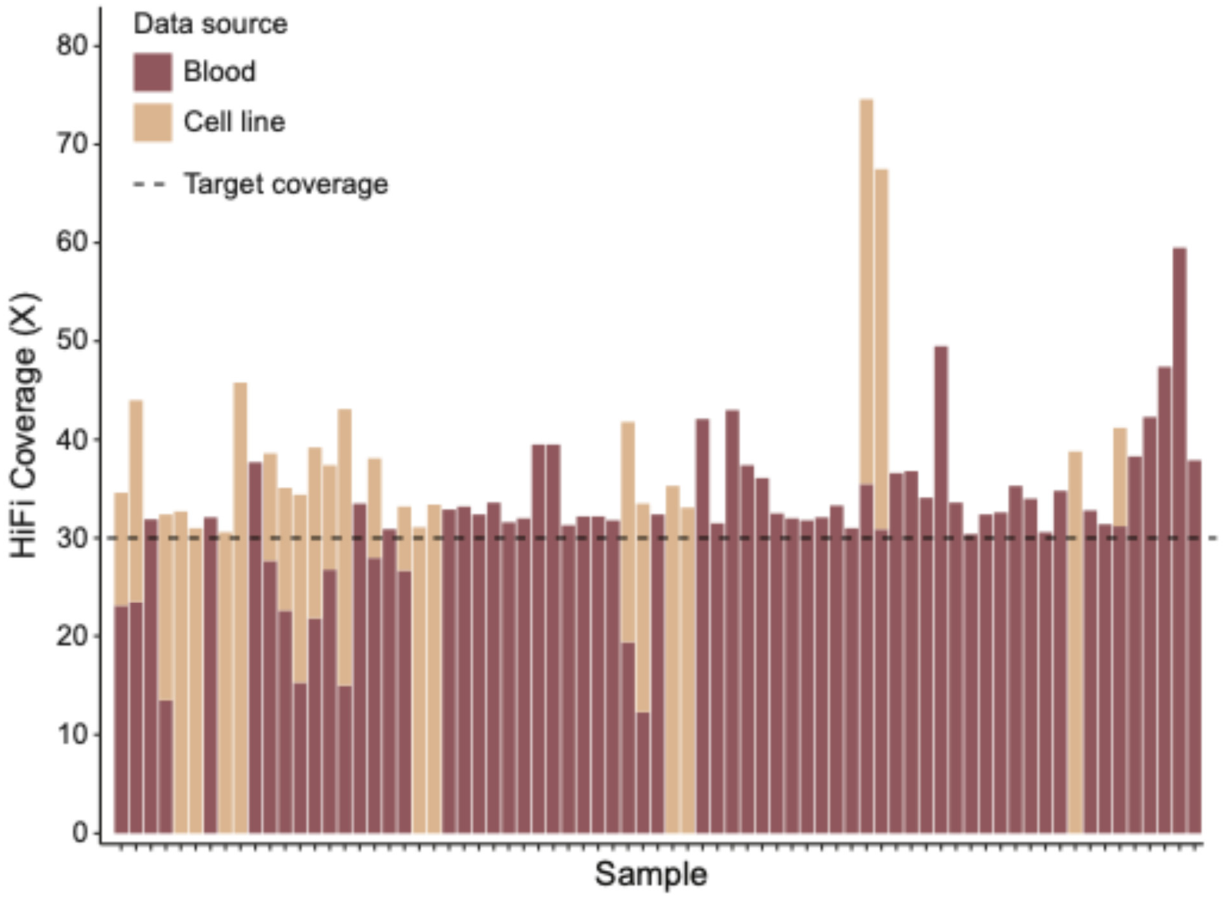

Supplementary Figure 2: HiFi sequencing depth by data source
HiFi sequencing depth across all n=73 samples in our dataset, sequenced to a target coverage of 30×. In total, 9 samples have HiFi data derived exclusively from cell lines, 47 samples are exclusively from peripheral blood, and the remaining 16 samples have a mixture of blood- and cell-line-derived HiFi data.

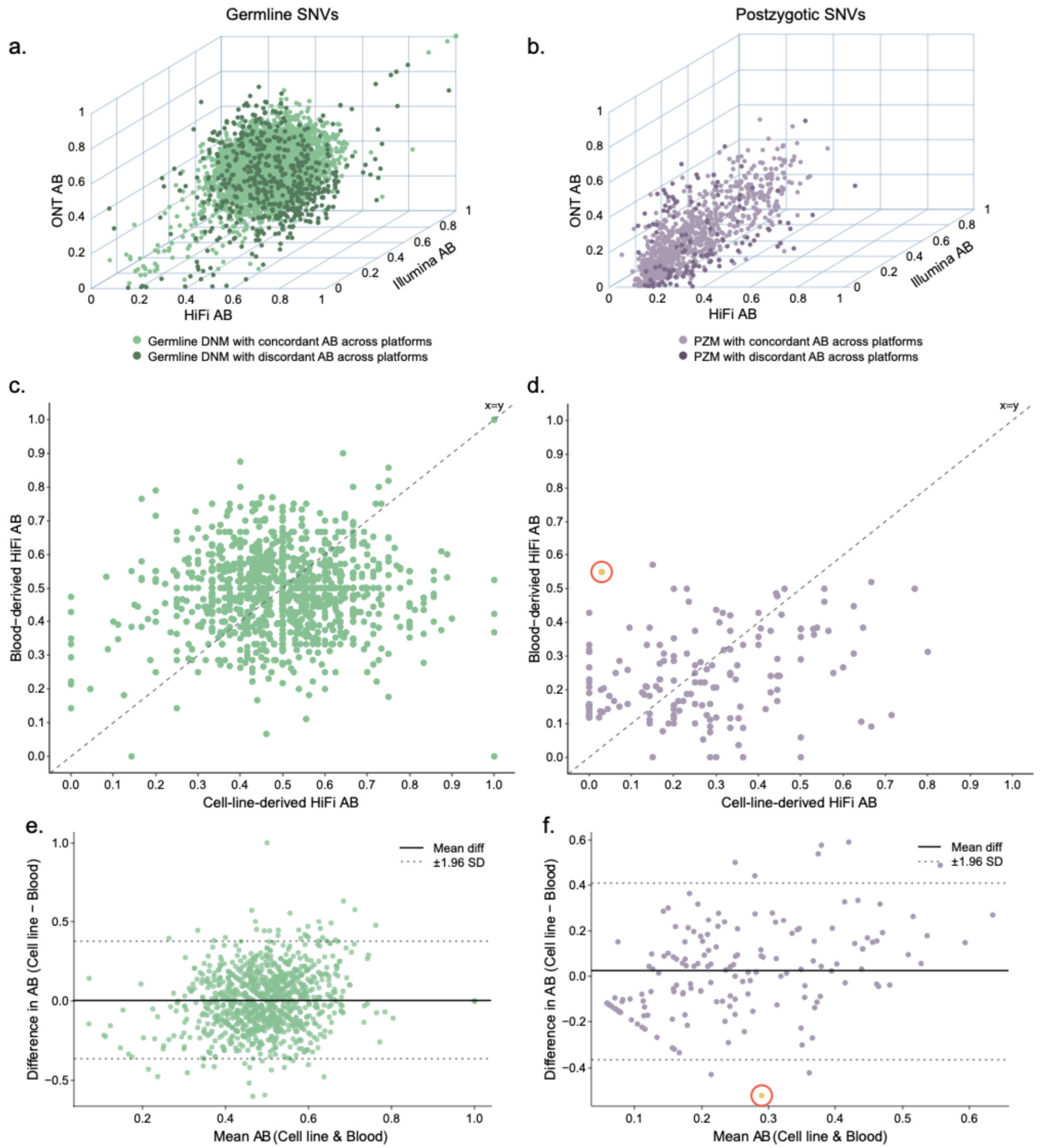

Supplementary Figure 3: Allele balance (AB) concordance scatterplots

a. AB of n=5,145 germline SNVs (DNMs) in PacBio HiFi, ONT, and Illumina read data. For each variant, a two-sided chi-squared test was used to determine whether its AB is concordant across platforms (p>0.05). A total of 89.1% of DNMs are concordant across platforms.
b. AB of n=917 postzygotic SNVs (PZMs) in the same sequencing read data as A. AB concordance was again defined by two-sided chi-squared test (p>0.05). A total of 85.7% of DNMs are concordant across platforms.
c. AB in cell-line- and blood-derived HiFi read data (restricted to reads with mapq>59) for n=979 germline SNVs from 15 samples. Although we do not see the expected x=y relationship between reads from both data sources, there is no evidence of systematic over- or underestimation

between sources. Each variant was tested for concordant AB using a Fisher’s exact test with Benjamini-Hochberg correction; none were significantly different ($p>0.05$).

d. AB in cell-line- and blood-derived HiFi read data (restricted to reads with mapq>59) for n=66 postzygotic SNVs from 15 samples. Similar to SNVs, we do not see a clear linear relationship between AB from data sources, but we also do not observe systematic bias. Each variant was tested for concordant AB using a Fisher’s exact test with Benjamini-Hochberg correction; highlighted in yellow and circled in red is the single variant with significantly different AB across sources ($p=0.0193$).
e. Bland-Altman plot showing the mean AB across data sources plotted against the difference between data sources for the same germline SNVs in (A). The mean difference in AB is 0.006, with a standard deviation of 0.188.
f. Bland-Altman plot showing the mean AB across data sources plotted against the difference between data sources for the same postzygotic SNVs in (B). The mean difference in AB is 0.022, with a standard deviation of 0.197.

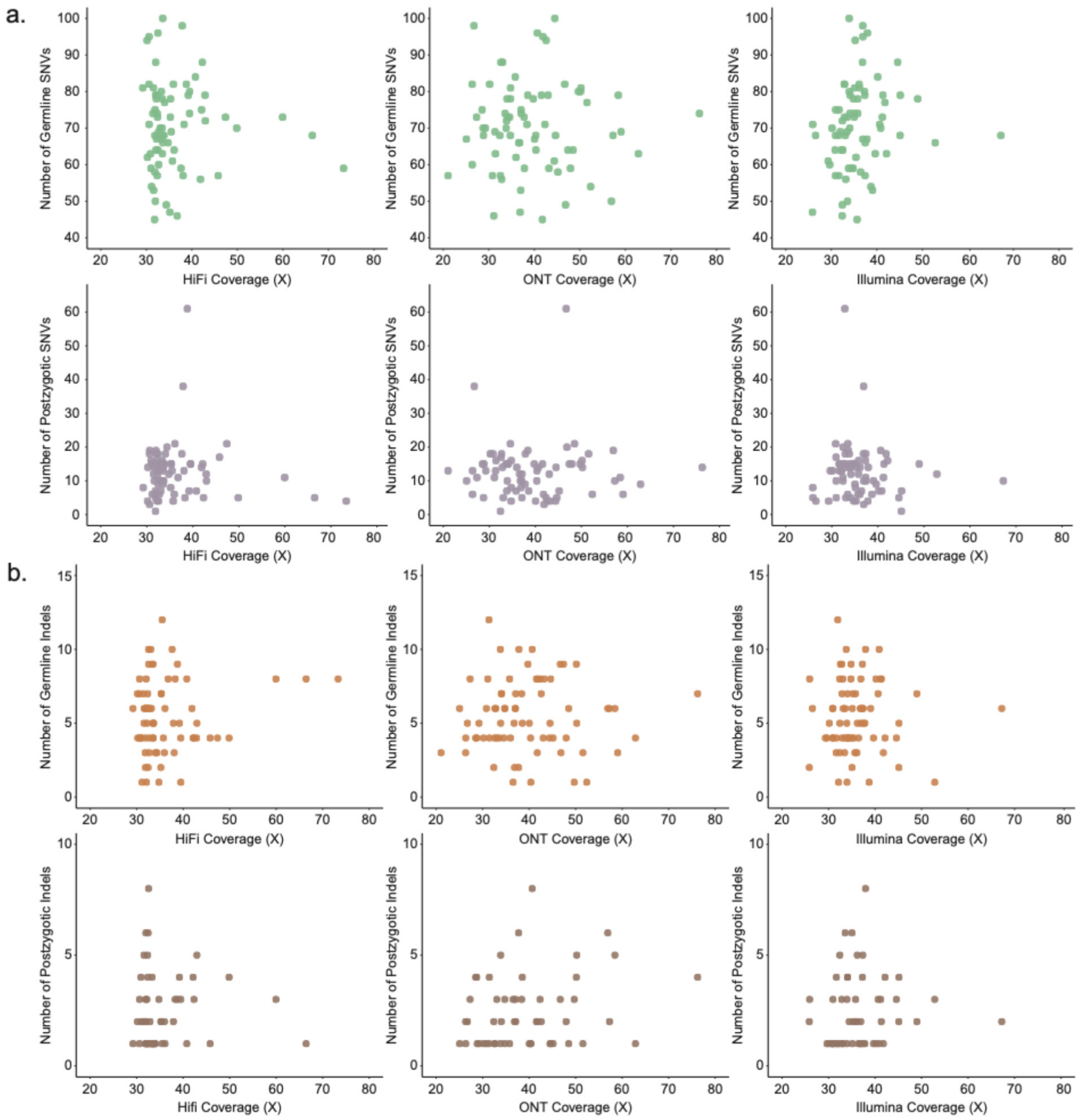

Supplementary Figure 4: Platform read depth versus *de novo* variant counts

a. The number of SNV calls plotted against the depth of coverage in each sequencing platform for n=73 samples. Based on negative binomial regression, we do not see a significant relationship between read depth and germline SNV count (p-values: HiFi 0.666, ONT 0.832, Illumina 0.128) or postzygotic SNV count (p-values: HiFi 0.463, ONT 0.917, Illumina 0.447).
b. The number of indel calls plotted against the depth of coverage in each sequencing platform for n=73 samples. Based on negative binomial regression, we do not see a significant relationship between read depth and germline SNV count (p-values: HiFi 0.253, ONT 0.944, Illumina 0.866) or postzygotic SNV count (p-values: HiFi 0.8925, ONT 0.0815, Illumina 0.698).

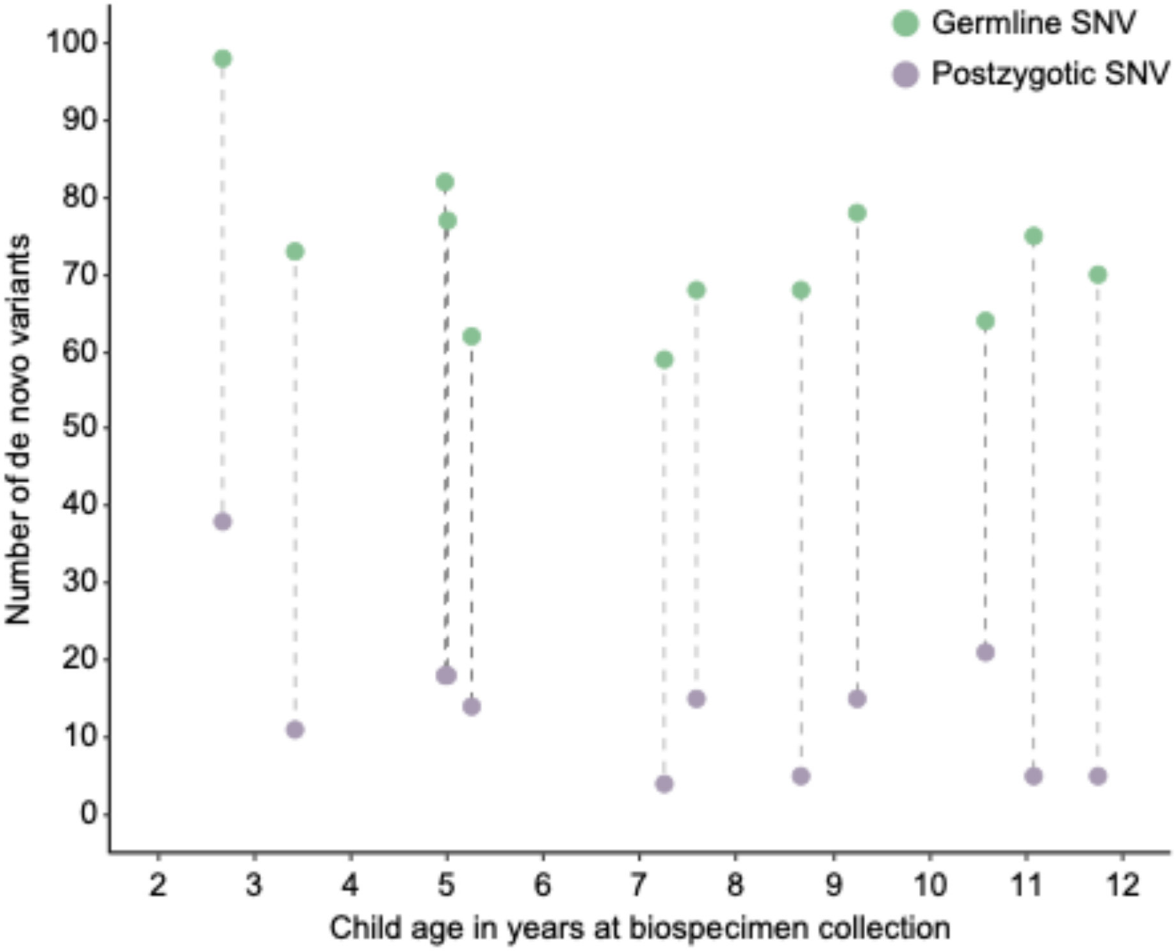

Supplementary Figure 5: Child age at sample collection and single-nucleotide substitution counts
The number of germline and postzygotic SNV calls in n=12 children, plotted against the sample age at time of biospecimen collection. By linear regression, we do not see a significant effect of age on the number of postzygotic SNVs (p=0.060). For germline SNVs, we incorporated paternal age into our regression model and found no significant effect of sample age at time of collection on the number of mutations (p=0.169).

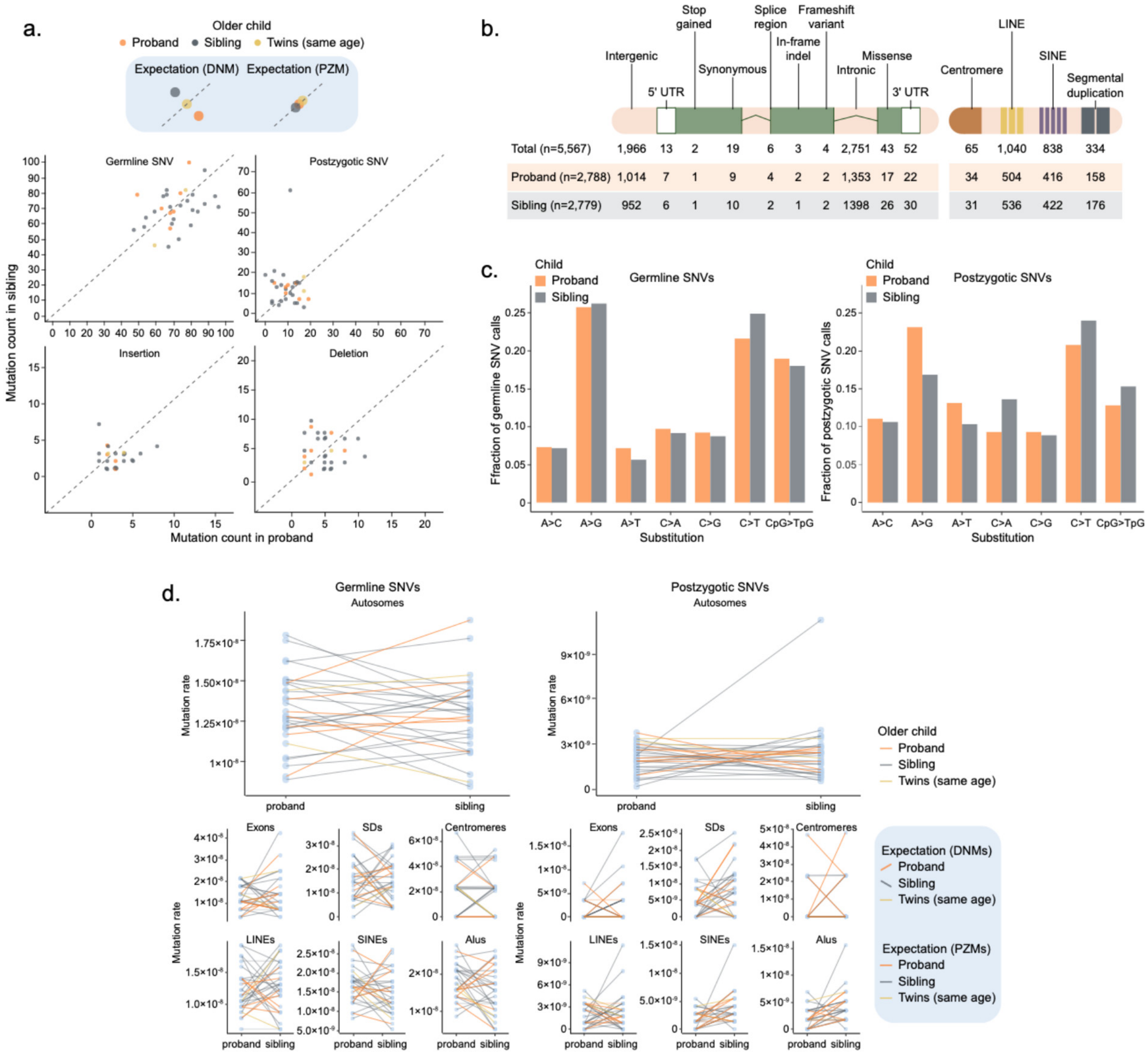

Supplementary Figure 6: Proband and sibling comparisons

a. The number of mutations observed in probands and siblings from 31 quads. Based on a linear regression accounting for paternal age at birth, we see no significant difference between counts in probands and siblings in any category (p-values: germline SNV 0.822, postzygotic SNV 0.301, insertion 0.184, deletion 0.919).
b. The germline and postzygotic single-nucleotide mutation spectrum in probands and siblings. Based on Benjamini-Hochberg corrected two-sided chi-squared tests, we see no significant difference for any mutation class (p-values: A>C 0.854, A>G 0.854, A>T 0.175, C>A 0.824, C>G 0.824, C>T 0.089, CpG>CpT 0.824).
c. The most severe predicted functional consequences for germline and postzygotic SNVs in probands and siblings (left), and the number of SNVs observed in repetitive regions of the genome (right).
d. The germline and postzygotic mutation rates in probands and siblings in different genomic regions. Probands and siblings from the same family are joined by lines indicating which child is older. Based on Benjamini-Hochberg corrected negative binomial regression with an offset (to account for paternal age at birth and the number of callable bases for each sample and region), we see no significant difference between probands and siblings in any region for germline SNVs ($p>0.99$ for each tested region) or postzygotic SNVS ($p>0.99$ for each tested region).

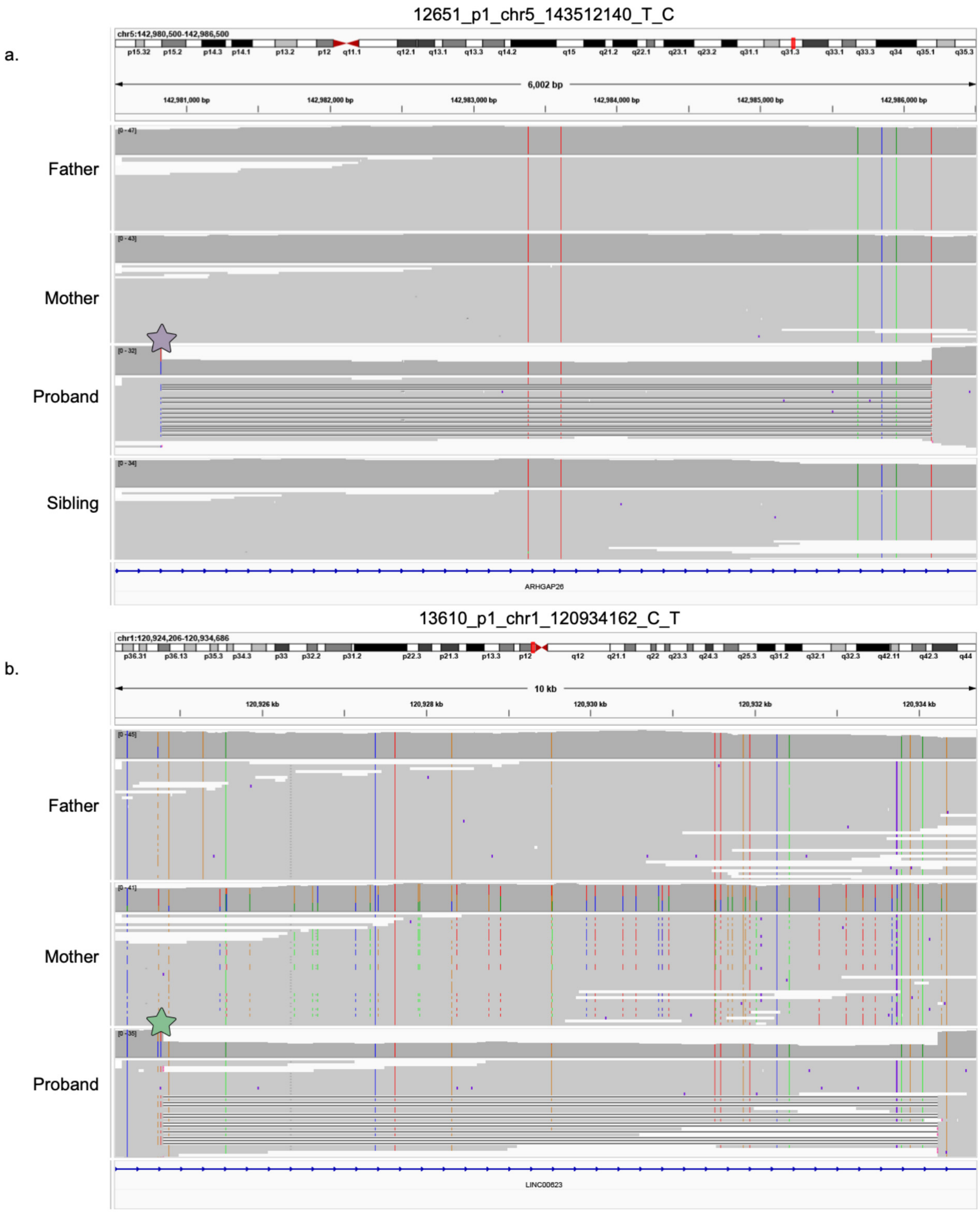

Supplementary Figure 7: *de novo* SNVs near *de novo* SVs

a. IGV screenshot of HiFi data aligned to GRCh38 for the members of family 12651 - a *de novo* T>C mutation can be seen immediately to the left of the SV. Note that this mutation appears to be germline in HiFi data but is imperfectly linked to surrounding SNPs in ONT data and was therefore deemed to be postzygotic in origin.
b. IGV screenshot of HiFi data aligned to GRCh38 for the members of family 13610 - a *de novo* C>T mutation can be seen immediately to the left of the SV.

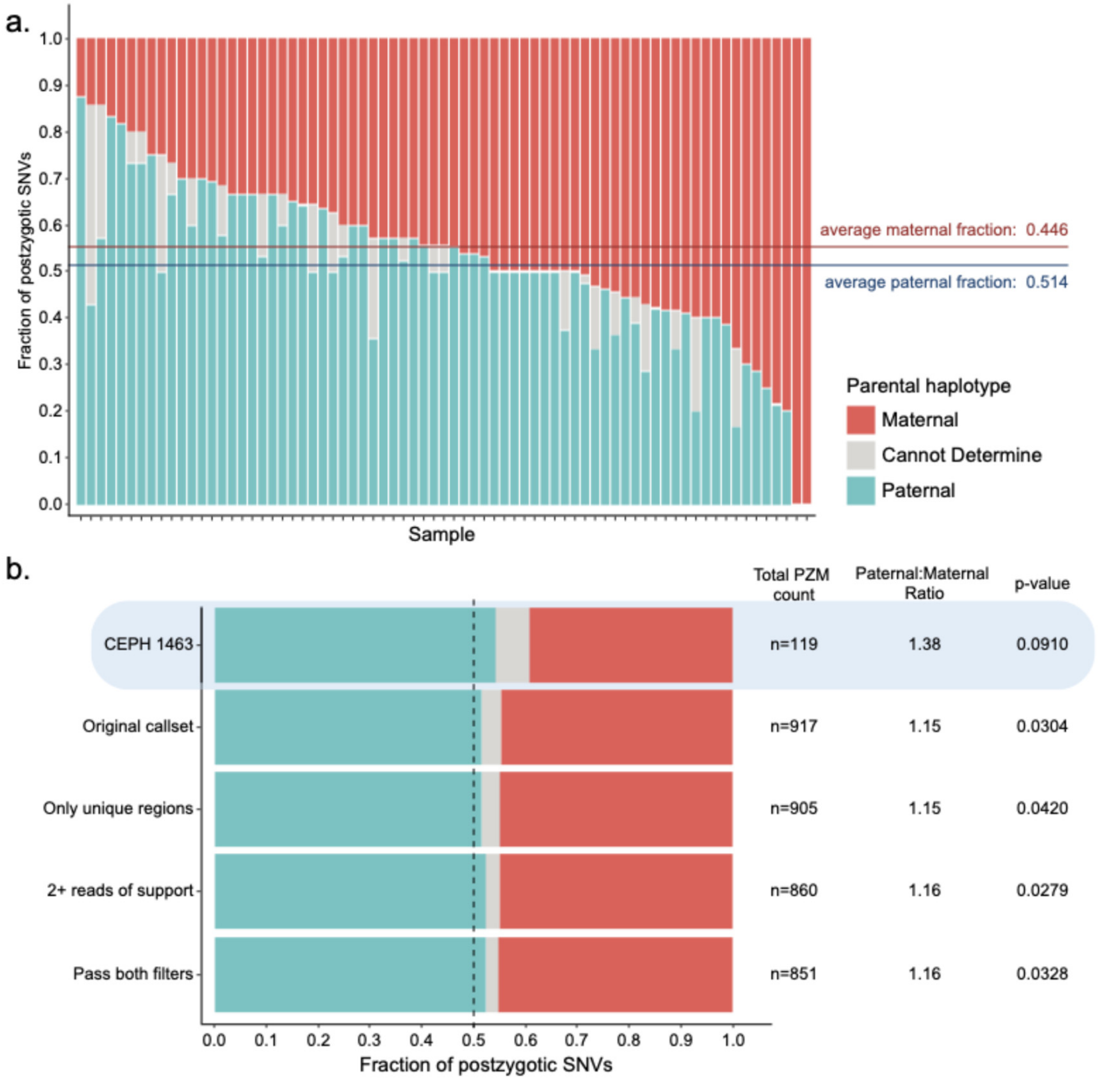

Supplementary Figure 8: Paternal bias in postzygotic SNVs

a. Paternal and maternal fractions of n=917 postzygotic SNVs across 73 samples.
b. Paternal and maternal fractions of CEPH 1463 (from Porubsky et al. 2024) compared to the final PZM callset with additional filters applied. Unique regions were defined as uniquely mappable with k-mer size 250 by Karimzadeh et al. 2018. P-values calculated using a two-sided Wilcoxon signed-rank test.

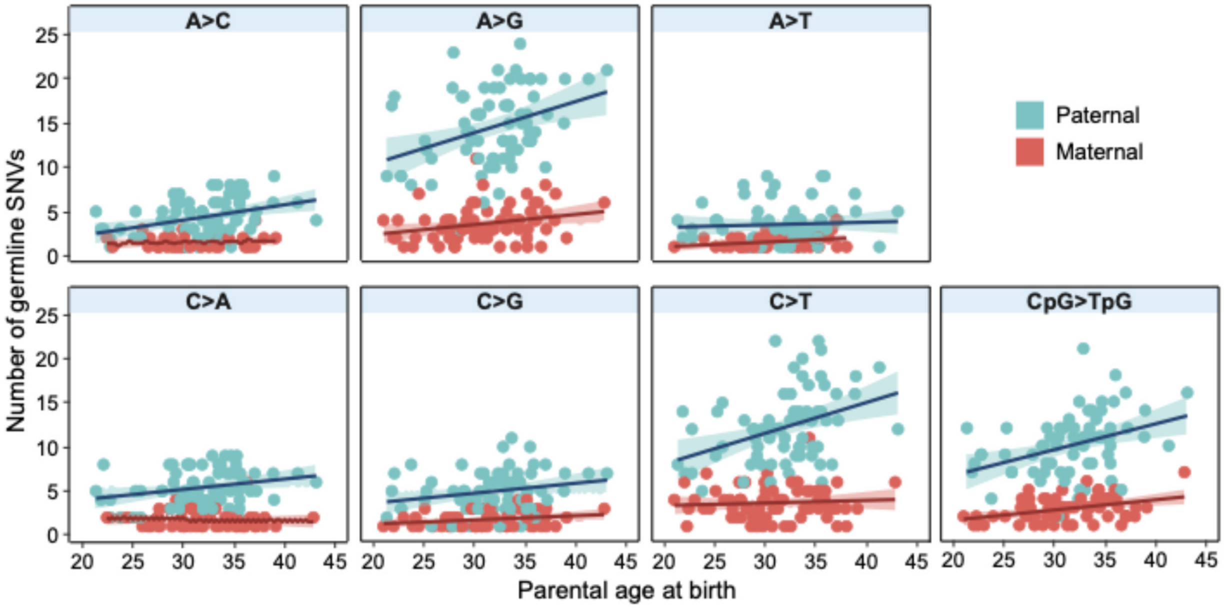

Supplementary Figure 9: Parental age effect by mutation class
The number of germline SNVs phased to maternal and paternal haplotypes plotted against each parent's age at birth. To estimate the age effect, we fitted a Poisson model using the glm function in R (glm(formula = mutation_count ~ parental_age, family = "poisson"). For significance values, we made a nested model with no age effect and found the log likelihood difference between both the full and nested model with a chi-squared approximation, as in Jonsson et al 2017. The number of paternal A>C, A>G, C>T, and CpG>TpG mutations are all significantly correlated with paternal age. Paternal p-values: A>C 0.00745, A>G 0.00644, A>T 0.251, C>A 0.159, C>G 0.537, C>T 0.00117, CpG>TpG 0.000141. Maternal p-values: A>C 0.693, A>G 0.0673, A>T 0.718, C>A 0.703, C>G 0.362, C>T 0.981, CpG>TpG 0.264.

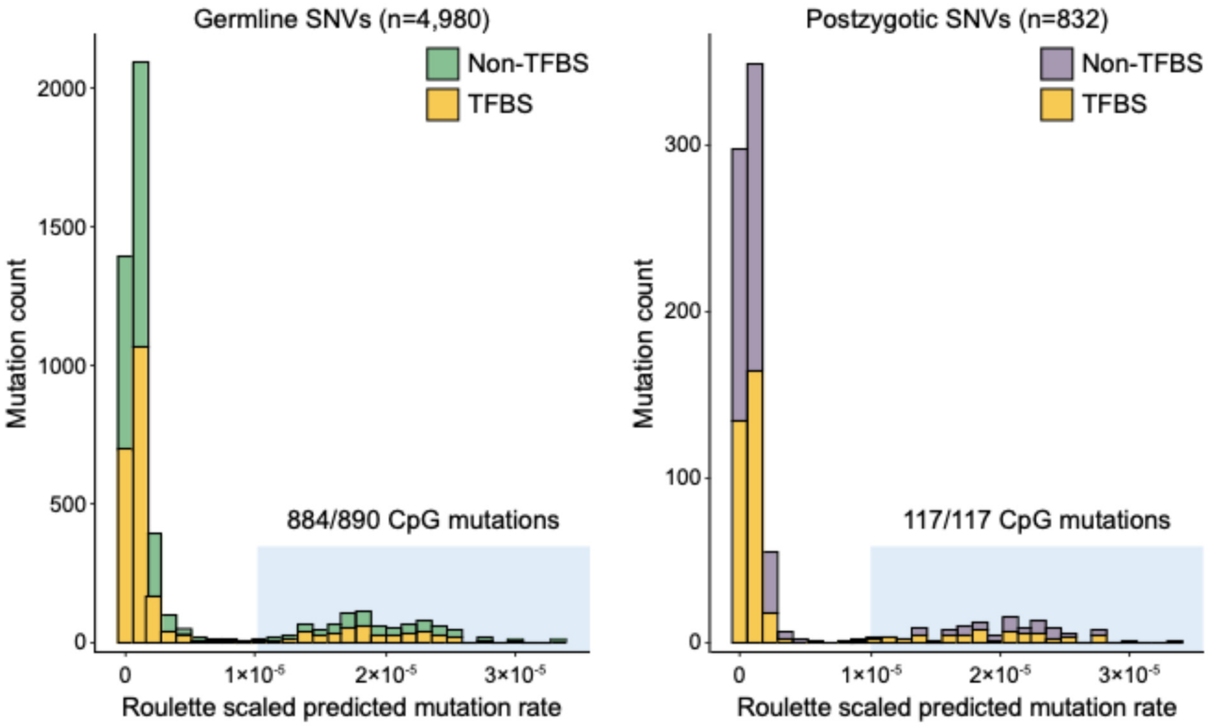

Supplementary Figure 10: Roulette-predicted mutation rates and TFBS
Roulette-predicted mutation rates for germline SNVS (left) and postzygotic SNVs (right) successfully lifted to GRCh38. Rates were scaled to our *de novo* callset, using gnomAD v2.1.1 synonymous variants as background sites. Variants are colored by presence/absence in transcription factor binding sites (TFBS). All but six mutations with rate>1e-5 are at CpG sites.

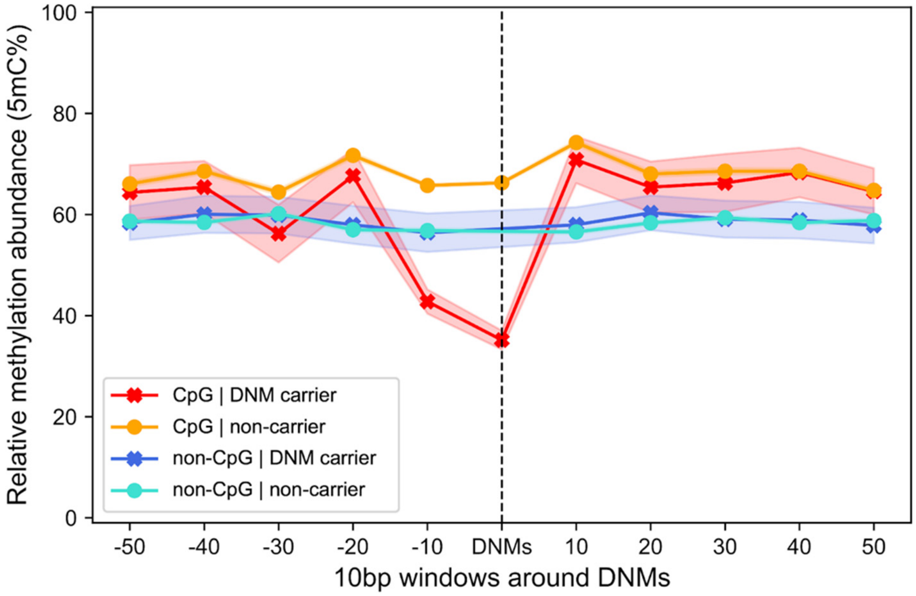

Supplementary Figure 11: Methylation abundance across ±50 bp surrounding 5,826 DNMs
Among the 6,062 DNMs initially identified in 41 probands and 32 unaffected siblings using the T2T-CHM13 reference from HiFi; 5,826 DNMs were retained after liftover to GRCh38 for methylation analyses. Mean methylation levels in 10 bp windows centered on each DNM (with a 1 bp window at the DNM site) were quantified using ONT-derived 5mC profiles from a subset of 24 probands and 26 unaffected siblings. Methylation level at each site was computed at the diploid level as the proportion of modified reads relative to the total reads, Nmod/(Nmod + Nother_mod + Ncanonical + Ndiff + Nnocall), where counts were obtained from Modkit (v0.3.1, https://github.com/nanoporetech/modkit). Of the original 5,826 DNMs, 1,147 (19.7%) occurred at CpG sites, with the remainder classified as non-CpGs. Curves represent the average methylation fraction across windows, stratified by CpG vs. non-CpG context and by sample type: DNM carriers are marked with crosses, and non-carriers with dots. Shaded regions indicate 95% confidence intervals, and the dashed vertical line marks the DNM-containing window (1 bp window).

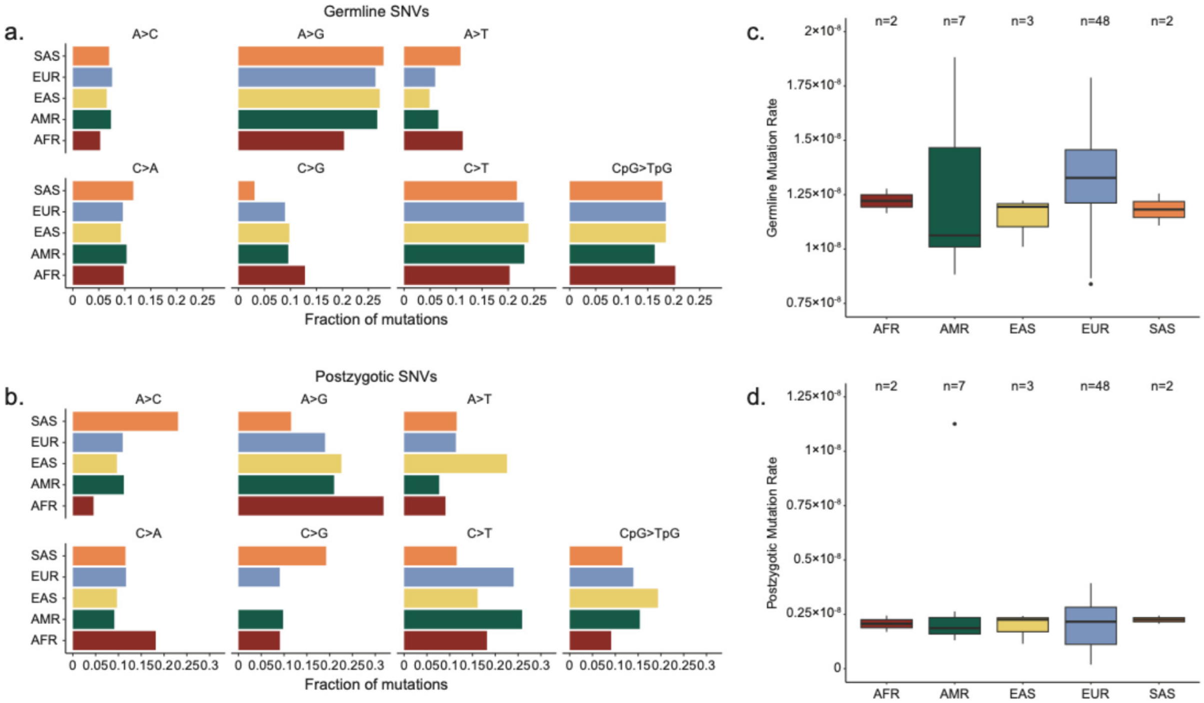

Supplementary Figure 12: Relationship between ancestry and single-nucleotide mutations

a. Germline single-nucleotide substitution spectrum using from each Somalier-defined ancestry group (AFR; African, AMR: Indigenous American, EAS: East Asian, EUR: European, SAS: South Asian). Based on a Benjami-Hochberg corrected chi-square test, we saw no significant relationship between mutation spectrum and European and non-European samples (p-values: A>C 0.891, A>G 0.891, A>T 0.683, C>A 0.891, C>G 0.891, C>T 0.891, CpG>CpT 0.891).
b. Postzygotic single-nucleotide substitution spectrum using the same ancestry groupings. Based on a Benjami-Hochberg corrected chi-square test, we saw no significant relationship between mutation spectrum and European and non-European samples (p-values: A>C 0.965, A>G 0.965, A>T 0.965, C>A 0.965, C>G 0.965, C>T 0.965, CpG>CpT 0.965).
c. Germline single-nucleotide substitution rates for samples using the same ancestry groupings. The center line defines the median value, and the box limits represent the upper and lower quartiles, the whiskers extend to the maximum and minimum points within 1.5× the interquartile range, and any points beyond are outliers. Based on an ANCOVA comparing European and non-European samples adjusted for both paternal and maternal age, ancestry has no significant effect on mutation rate (p=0.781).
d. Postzygotic single-nucleotide substitution using the same ancestry groupings. The center line defines the median value, and the box limits represent the upper and lower quartiles, the whiskers extend to the maximum and minimum points within 1.5× the interquartile range, and any points beyond are outliers. Based on the same ANCOVA analysis, ancestry has no significant effect on mutation rate (p=0.159).

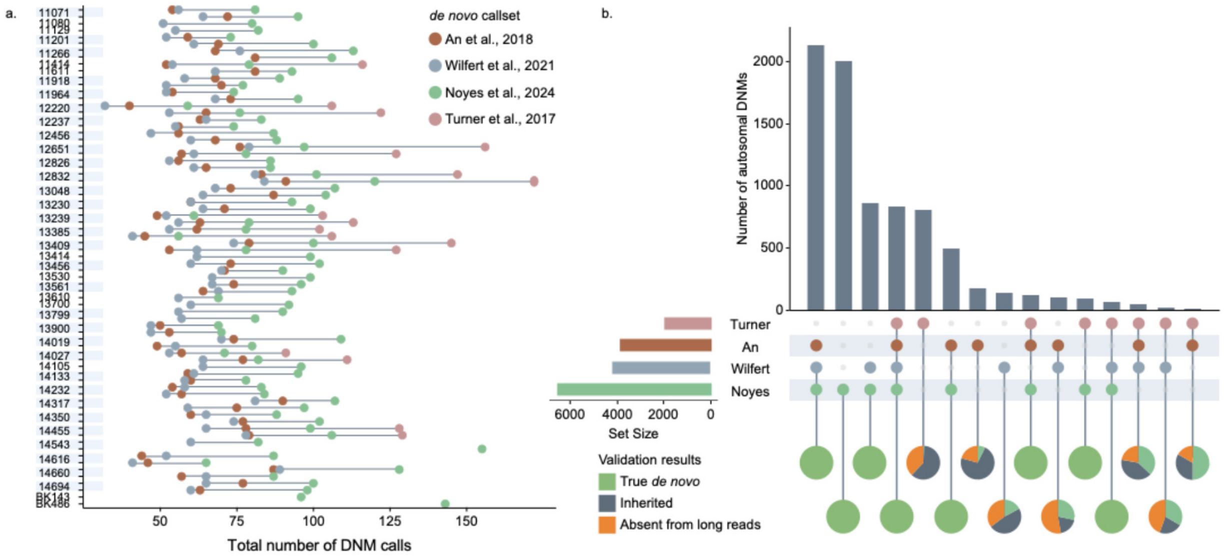

Supplementary Figure 13: Comparison to other studies

a. DNM counts for all 73 samples across previous studies. We identified more DNMs per sample than most previous Illumina-based studies, with the exception of Turner et al. Siblings are grouped together and highlighted by a blue box.
b. DNMs identified by multiple studies have the highest true positive rates, while DNMs exclusive to a single study tend to be false positives.

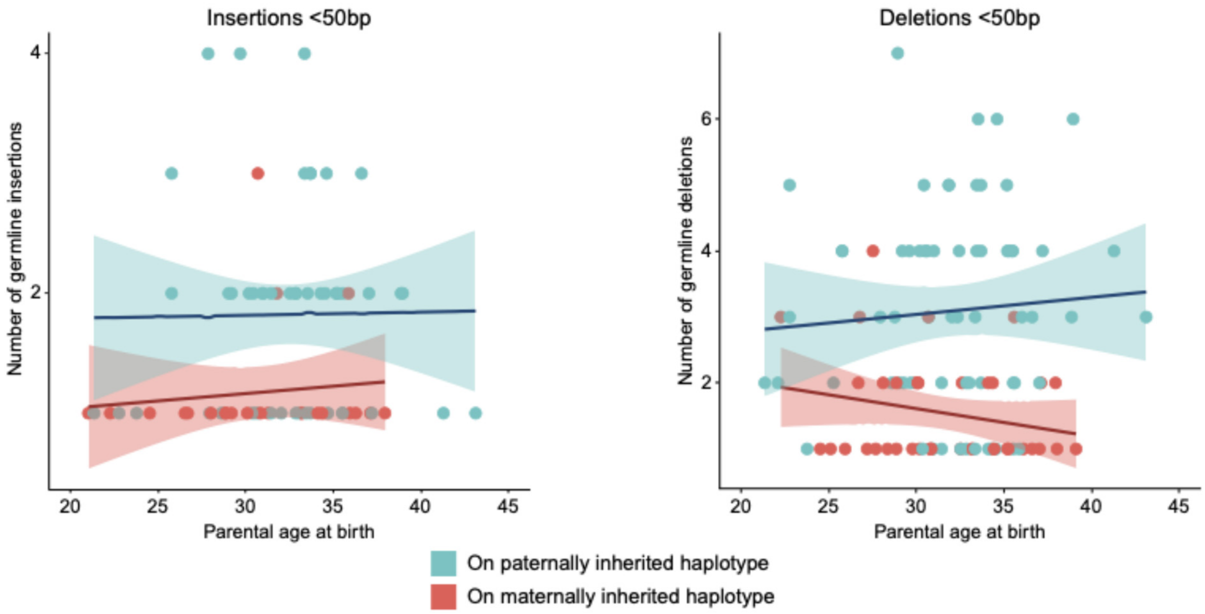

Supplementary Figure 14: Indel parental age effect

Indel counts by parental age for n=73 samples. Neither insertions nor deletions are significantly correlated with year of parental age by linear regression (p-values: paternal insertions 0.85, maternal insertions 0.60, paternal deletions 0.63, maternal deletions 0.16).

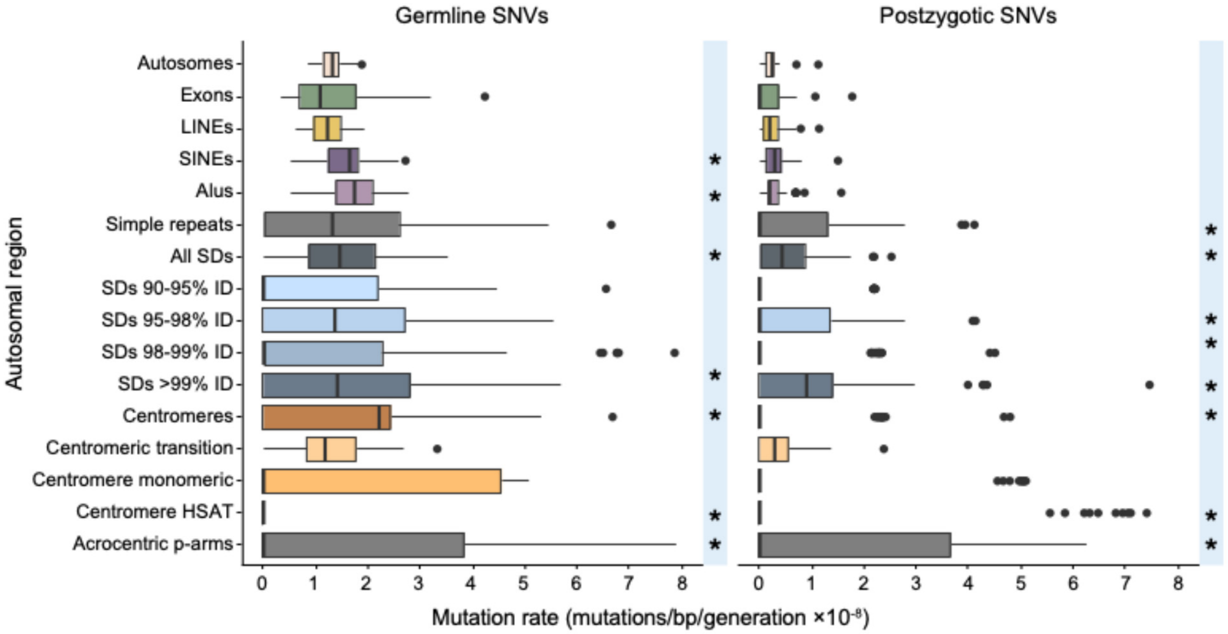

Supplementary Figure 15: Mutation rates in segmental duplications and centromeric regions

The germline (left) and postzygotic (right) single-nucleotide mutation rates across repetitive regions for n=73 samples. Segmental duplications (SDs) are stratified by percent identity (% ID). The center line defines the median value, and the box limits represent the upper and lower quartiles, the whiskers extend to the maximum and minimum points within 1.5× the interquartile range, and any points beyond are outliers. Asterisks indicate a region is enriched for (either germline or postzygotic) mutations relative to the autosomes as a whole, as calculated with a Welch two-sample t-test with Benjamini-Hochberg correction. P-values for germline SNVs: exons 0.73, LINEs 0.36, SINEs 0.00025, Alus 0.000011, Simple repeats 0.25, All SDs 0.045, SDs 90-95% ID 0.38, SDs 95-98% ID 0.16, SDs 98-99% ID 0.33, SDs >99% ID 0.092, Centromeres 0.016, Centromeric transition 0.73, Centromere monomeric 0.058, Centromere HSAT 0.011, Acrocentric p-arms 0.0085. P-values for postzygotic SNVs: exons 0.24, LINEs 0.96, SINEs 0.24, Alus 0.064, Simple repeats 7.7e-5, All SDs 1.91e-6, SDs 90-95% ID 0.96, SDs 95-98% ID 0.048, SDs 98-99% ID 0.037, SDs >99% ID 2.9e-5, Centromeres 0.037, Centromeric transition 0.064, Centromere monomeric 0.053, Centromere HSAT 0.0019, acrocentric p-arms 7.7e-8.
