# Supplementary material for: Long-read sequencing of families reveals increased germline and postzygotic mutation rates in repetitive DNA
Source: Nat Commun. 2026 Mar 9;17:3717. doi: 10.1038/s41467-026-70342-1 (PMC13102968; doi:10.1038/s41467-026-70342-1)
Supplement: Supplementary file 2 — Description of Additional Supplementary Files [file 41467_2026_70342_MOESM2_ESM.pdf]

## Description of Additional Supplementary Files

Supplementary Data 1: All DNMs in callset

Supplementary Data 2: Functional annotations of DNM calls

Supplementary Data 3: *de novo* SVs identified by Sui et al.

Supplementary Data 4: All parent-of-origin assignments counts for germline and postzygotic SNVs

Supplementary Data 5: gnomADv4.1 frequencies of germline and postzygotic SNVs lifted to GRCh38
